# Remarks on sentinel node biopsy in head and neck cancer

**DOI:** 10.1038/sj.bjc.6602293

**Published:** 2004-12-14

**Authors:** A F Kovács

**Affiliations:** 1 Universitatsklinik Haus 21 Klinik und Poliklinik fur Kiefer- und Plastische Gesichtschirurgie, Theodor-Stern-Kai 7, Frankfurt am Main D-60590, Germany

**Sir**,

The status of the regional lymphatics is one of the most important prognostic parameters in patients with head and neck cancer, and the presence or absence, level, and size of metastatic neck disease are crucial for treatment and survival. Due to the limited sensitivity and specificity of the usual diagnostic tools like ultrasound, CT, MRI, and PET (Stuckensen *et al*, 2000), a pathohistological staging of the neck was generally adopted to remove and detect occult metastases, which could not be detected by these imaging techniques. A large number of elective neck dissections (ND) where the pathohistological examination of the surgical neck specimen did not reveal any positive nodes was accepted. This surgical procedure was associated with risks and morbidity of the patients concerned.

In the last years, beginning with a case report by Alex and Krag (1996), sentinel node biopsy (SNB) in head and neck cancer became a very interesting field of clinical investigation. Other investigators followed, and the recent article of Höft *et al* (2004) mentioned some of them. Because the method of SNB was interlaced with the N0 neck, that is, a neck without clinically detectable nodal metastasis, the procedure generally adopted by investigators like Höft *et al* (2004) was to carry out an elective ND parallely to SNB. The pathohistological results were compared, with the sentinel nodes carrying metastasis being the false-negative results of the imaging techniques. Experience still is limited, follow-up too short. We felt, however, that Höft *et al* (2004) did not discuss some relevant topics in depth.

The first problem is the definition of the N0 neck, in other words the method of examination on which the clinical diagnosis was based. Höft *et al* (2004) based staging of the neck on ultrasound examination and had a rate of 24% positive sentinel nodes in 50 patients. Contributors to the first multicentre study on sentinel node biopsy in head and neck cancer (Ross *et al*, 2004) based their staging on either clinical palpation or radiological imaging techniques like CT and had an upstaging rate of 34% in 134 patients. Kovács *et al* (2001, 2004a, 2004c) had an upstaging rate of 5-15% following neck staging based on PET in 15 and 38 patients. Thus, SNB reflected the accuracy of the clinical and radiological staging methods, and the ideal diagnostic prerequisite for SNB is not yet found.

The second problem is the dimension of the primary. Höft *et al* (2004) stated that ‘if a complete peritumoral injection of the tracer is not possible, the patient is not eligible for the sentinel node method’. We agree that peritumoral accessibility is more important than T classification, but large T3 and T4 primaries pose problems due to destroyed lymphatic drainage. Local intra-arterial induction chemotherapy, however, did not seem to alter lymphatic drainage (Kovács *et al*, 2004b) and might be a modality of treatment that can be added prior to SNB reducing tumour size.

The third and main problem is the omission of an elective ND, which would potentially achieve a benefit for the patient concerning risk, morbidity, and life quality. This would depend on the reliability of SNB. In the study of Höft *et al* (2004), ‘no patient with tumor-free sentinel nodes was found to have a metastasis in a nonsentinel lymph node’. False-negative results have been very rare in all previous studies, too. This would encourage the omission of elective ND in favour of SNB. However, Höft *et al* (2004) falsely stated that ‘So far, only Ross *et al* (2002) have reported on a study of a true biopsy of the sentinel lymph node without elective neck dissection’. Kovács *et al* (2001) reported on true biopsy of the sentinel node without elective ND, and all consecutive patients have been treated that way (Kovács *et al*, 2001, 2004a, 2004b and 2004c). Diagnostics using PET in combination with SNB considerably reduced the number of elective ND, and the inconspicuous follow-up time of 80 patients to date surpassing a median of 2 years makes it not likely that this will be paired with hazard. Some contributors of the mentioned multicentre study also adopted this procedure, and there is hope that SNB without elective ND will be the staging procedure of the future in a large number of head and neck cancer patients.
